# Oral Facial Manifestations of Sanjad–Sakati Syndrome: A Literature Review

**DOI:** 10.3390/children9040448

**Published:** 2022-03-22

**Authors:** Sara Alghamdi

**Affiliations:** Department of Preventive Dental Science, College of Dentistry, Majmaah University, Al-Majmaah 11952, Saudi Arabia; sa.mohammed@mu.edu.sa

**Keywords:** dental manifestations, dental management, Sanjad–Sakati syndrome, general ansthesia

## Abstract

Aim: To perform a comprehensive review of orofacial manifestations of Sanjad–Sakati syndrome (SSS). Methods: A comprehensive electronic literature search was performed using PubMed, Scopus and Cochrane library databases. The search keywords included were “Sanjad–Sakati syndrome (SSS)”, “dental manifestations”, “dental management”, “oral health”, “dental care for patients with SSS”, “dental health of people with SSS”, “caries”, and “oral hygiene”. The inclusion criteria were papers published only in English, papers published by August 2021, and papers discussing orofacial manifestations of SSS and language. Results: The search of the databases retrieved eleven case reports and three case series studies. Overall, 56 cases (11 case reports and 3 case series studies) were reported on Sanjad–Sakati syndrome in the published literature. The majority of the reports are from the Middle Eastern region. Conclusions: The reported orofacial manifestations of SSS include beaked nose, depressed nasal bridge, enamel hypoplasia, hypodontia, low-set ears, posteriorly rotated ears, deep-set eyes, microcephaly, microdontia, micrognathia, prominent forehead, retrognathia, and thin lips. The review paper also establishes the importance of the dental under general anesthesia in SSS individuals.

## 1. Introduction

Sanjad–Sakati syndrome (SSS) is an autosomal recessive disorder reported three decades ago by Sanjad and co-workers [1] in 12 infants (6 boys, 6 girls) of age ranges from 1 day to 15 months. The majority of the initial reports on SSS were reported in the Middle East and North Africa, and recently in India. The highest incidence was reported in Kuwait, Oman, Bahrain, Jordan, Saudi Arabia, Sudan, Tunisia, Palestine, Iran, and Egypt [1,2,3,4,5,6,7,8,9,10,11,12]. This syndrome occurrence is caused by a mutation in the TBCE (Tubulin Folding Cofactor E) gene on chromosome 1q42-43 [3]. The exact incidence of SSS in Saudi Arabia is unknown; however, its estimated incidence is 1 per 40,000–100,000 live births [2]. The characteristics of SSS include severe growth failure, congenital hypoparathyroidism, dysmorphism, and learning disabilities [1]. Pharmacological management and treatment for SSS mainly include calcium and vitamin D (calcitriol) supplements [3,6]. SSS can affect oral and dental health for multiple reasons, including: neglected oral hygiene, high caries incidence due to a lack of growth and development of the masticatory apparatus, continuous need for soft and semisolid food, nursing bottles, and sugary medications that are consumed during recurrent infections [13]. Permanent congenital hypoparathyroidism is common to DiGeorge syndrome (DS), Kenny–Caffey syndrome (KCS), and SSS [1,3,6]. SSS may be confused with KCS Type 1, because of its mutation in the TBCE gene and the fact that it shares similar phenotypic traits. However, unlike SSS, KCS Type 1 presents with osteosclerosis, medullary stenosis of the long bones, and normal intelligence [3,6]. SSS may also be confused with DS because this syndrome is characterized by congenital cardiac anomalies, congenital hypoparathyroidism, T-cell immunodeficiency, and dysmorphic facial features [7,14]. However, in SSS, the dysmorphic features are different, and severe growth failure occurs both in utero and postnatal [14]. Superior mesenteric artery syndrome due to severe growth retardation rarely manifests in SSS, with an incidence of (0.1–0.3%) [15]. Visceral myopathy is another rare disabling condition in SSS that results from chronic intestinal pseudo-obstruction [16]. Various reports and case studies regarding SSS were reported in the literature. However, orofacial manifestations of SSS were not discussed properly to date. Moreover, most previous publications are case reports, with a limited number of review articles. Therefore, this study aimed to perform a comprehensive review of the oral manifestations and difficulties of SSS, and the complexities of the dental management of individuals with SSS.

## 2. Methodology

**Figure 1:**
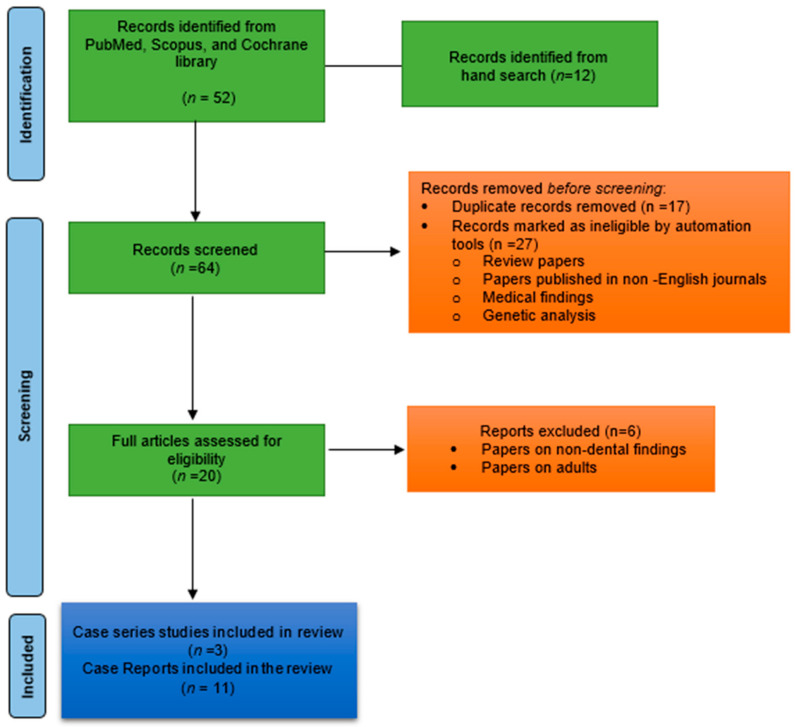
PRISMA 2020 flow diagram used in the review process.

This review performed a comprehensive electronic search of the literature in PubMed, Scopus, and the Cochrane library databases. The search keywords included were “Sanjad–Sakati syndrome”, “dental manifestations”, “dental management”, “oral health”, “dental care for patients with SSS”, “dental health of people with SSS”, “caries”, and “oral hygiene”. The inclusion criteria were papers published only in English, papers published by August 2021, and papers discussing orofacial manifestations of SSS. The exclusion criteria include discussion of papers other than dental manifestations (medical, genetic, and neurological), review, and systematic reviews. This literature review was accomplished to define the orofacial manifestations of Sanjad–Sakati syndrome. This review differs from a systematic review in that it comprises a general discussion of individuals with SSS and a hypothesis was not stated. The retrieved articles were carefully read to help in a further search for additional articles. The search is illustrated in Figure 1.

## 3. Results

The search of three databases yielded eleven case reports [6,7,10,13,16,17,18,19,20,21,22] and three studies [1,3,23]. The retried case reports are summarized in Table 1 and the case series studies are outlined in Table 2. The majority of the reports are from the Middle Eastern region. Overall, fifty-six cases were reported on Sanjad–Sakati syndrome in the published literature. Among them, nineteen were males and twenty-six were females; Naguib et al. [23] reported from Kuwait but did not report the sex of the patients. Fifteen subjects were from Saudi Arabia, twenty-one subjects from Kuwait, eight from the United Kingdom, three cases from Egypt, two from Oman, and one each from India, Morocco, Jordon, and Israel. The reported orofacial manifestations include thin lips, beaked nose tip, deep-set eyes, microcephaly, depressed nasal bridge, micrognathia, smooth filtrum, low-set ears, posteriorly rotated ears, prominent forehead, hypodontia, microdontia, retrognathia, and enamel hypoplasia. The Sanjad–Sakati syndrome key features from the reported cases are described below.

### 3.1. Etiology

SSS is an autosomal recessive syndrome mainly seen in the offspring of phenotypically normal consanguineous parents and primarily affects siblings [23]. The syndrome is caused due to mutations in the TBCE gene located in the chromosome region 1q42-q43 [24,25]. Sequencing analysis of the TBCE gene in SSS individuals revealed a homozygous deletion (12-bp) [26].

### 3.2. Neonatal Period

During the neonatal period, metabolic disturbances may lead to a number of pathological manifestations in SSS patients, including hypocalcemia, hyperphosphatemia, hypomagnesemia, and permanent inherent hypoparathyroidism [27]. These metabolic disturbances play a crucial role in developing nephrocalcinosis, medullary stenosis of the long bones, convulsions, and seizures [27]. SSS also increases the risk of respiratory infections, stunted growth, mental retardation, and pathological bone fractures [18]. Several measures can be taken to prevent these fractures, such as ensuring the intake of mineral-supplemented milk and delicate handling of the limbs, particularly when lifting and placing intravenous lines [27]. Pal et al. [28] reported a rare chronic intestinal pseudo-obstruction that leads to visceral myopathy complicated by intestinal failure, sepsis, and early mortality in the neonatal period. In such cases, a radiological survey of the skeleton and a bone densitometry examination are recommended [27].

### 3.3. Phenotypic and Endocrinological Features

The commonly observed dysmorphic features in newborns with SSS are prominent forehead, deep-set eyes, depressed nasal bridge, long philtrum, thin upper lip, short stature, micrognathia, blue sclera, and small hands and feet [3,6,24,29]. Ophthalmic manifestations in children include microphthalmia, corneal opacities, errors of refraction, strabismus, and retinal vascular tortuosity [3,30,31]. A rare ocular manifestation of bilateral congenital corneal clouding has also been reported [25]. The unique classical endocrinological features of SSS are primary hypoparathyroidism, growth hormone insufficiency, and hypocortisolemia [32].

### 3.4. Neurological Features

Neurological manifestations of SSS are microcephaly, developmental delay, mental retardation, hypocalcemic tetany, hyperphosphatemia, repeated attacks of symptomatic seizures, and craniofacial deformities [14,32,33]. Brain imaging using magnetic resonance imaging (MRI) and computerized tomography (CT) suggests identifying severe hypoplasia of the anterior pituitary and corpus callosum and intracranial calcifications. Decreased white matter bulk was reported in 30% of the individuals with SSS [32]. A rarely reported MRI finding in SSS is partial agenesis of the corpus callosum [15]. Status epilepticus has also been rarely reported [34].

### 3.5. Otolaryngologic Features

Children with SSS may exhibit recurrent ear infections, severe respiratory insufficiency, upper respiratory infections, obstructive sleep apnea, scoliosis, redundant supraglottic mucosa, retroflexed epiglottis, mandibular hypoplasia, and retrognathism [35]. These features may lead to severe medical conditions that require exceptional airway management during general anesthesia administration in these patients [36].

### 3.6. Medical Management

Medical management and treatment for SSS include administering calcium and vitamin D (calcitriol) supplements [3,6,23,32,37]. Growth hormone (GH) insufficiency in SSS patients was treated with GH therapy; however, GH therapy was reported to elicit no response in some cases [7,38]. Levothyroxine was considered as a treatment for hypothyroidism in these individuals [39]. A patient suffering from macrocytic anemia was treated with oral folic acid supplements as the patient suffered from allergies to cow’s milk proteins and folic acid deficiency [40]. Another case report described a patient needing aggressive nutritional support via gastrostomy tube [5]. SSS patients typically suffer from recurrent infections, which may progress to symptomatic hypocalcemia, necessitating increased doses of calcium supplements and alfacalcidol [41]. Close monitoring is required following the resolution of the infections, with timely reduction of the active analogs of vitamin D and calcium supplements to prevent hypercalciuria, hypercalcemia, and nephrocalcinosis [42,43]. Anteet et al. [44] strongly recommend routine evaluation of thyroid function and autoimmune antibodies during the follow-up in one-third of individuals with SSS.

### 3.7. Dental Manifestations

Frequently reported dental manifestations of SSS are: delayed teething, dental caries, abnormal tooth shape, high-vaulted palate, micrognathia, supernumerary maxillary right lateral deciduous incisors, microdontia, oligodontia, deep overbite, and increased overjet [1,3,6,7,10,13,16,17,18,19,20,21,22,23]. The reported facial findings include a beaked nose, deep-set eyes, depressed nasal bridge, floppy ear lobes, frontal bossing, large and low-set years, long philtrum, microcephaly, and thin upper lip [1,3,6,7,10,13,16,17,18,19,20,21,22,23]. Low parathyroid hormone levels, which lead to hypocalcemia, were also proposed as the etiology of enamel hypoplasia and enamel opacities [39]. Studies [14,41] reported that these abnormalities could occur in children who suffer from hypocalcemia during the period of enamel formation and were observed more often in the permanent than the primary dentition. In addition, failure of primary tooth eruption may occur due to a mutation in the parathyroid hormone 1 receptor (PTH1R) [41]. Enamel hypoplasia is a defect causing reduced enamel thickness and results in severe dental caries in SSS patients [14,34]. Retarded growth of the masticatory apparatus renders these patients in continuous need of soft and semisolid food, nursing bottles, sugary medications consumed during recurrent infections, and neglected oral hygiene, which act as a predisposing factor for dental caries [13,44].

### 3.8. Dental Management

In a few cases, dental treatment for SSS can be performed under local anesthesia. Local anesthetics should be used cautiously, especially Bupivacaine, to avoid the risk of cardiotoxicity due to hypocalcemia in the SSS population [45]. In most reported cases, dental management is performed under general anesthesia. Wasersprung et al. [15] described a child’s dental rehabilitation with SSS under general anesthesia, without serious medical complications other than a desaturation event (80% SaO_2_), treated with bronchodilators ventilation [46]. Al-Malik [19] reported the uneventful anesthetic management of one SSS case, and El Batawi [13] reported that a child with SSS required two general anesthesia sessions for dental treatment. Prophylactic antibiotics were prescribed to prevent chest infection, and the tube in the second case was selected according to the child’s weight, rather than age. In both cases, serious medical issues did not occur, and admission to the intensive care unit was not required [13,25]. Hassona et al. [21] reported dental treatment performed under local anesthesia on a 15-year-old patient who cooperated well with the dental team. In all these cases, teeth were restored using stainless steel crowns, due to their longevity. Waserprung et al. [15] reported a patient with an anodontia of 12 permanent teeth diagnosed radiographically, and Hassona et al. [21] reported multiple missing teeth in a patient. However, El Batawi [13] and Al-Malik [16] both reported that radiographic examination was not possible in their cases. Hence, the possibility of anodontia in their patients cannot be excluded. El Batawi [13] also encountered difficulties in performing stainless steel crowns for microdontia first primary molars in an Arabian child. He used upper, opposite-side crowns to restore the mandibular primary molars, due to the smaller mesiodistal width of maxillary molars [13]. Management of SSS patients under general anesthesia depends on appropriate preoperative assessment and evaluation. Preoperative pulmonary function tests, along with chest radiographs, are recommended, due to recurrent respiratory infections in SSS individuals [47,48]. Polysomnography is suggested to confirm central hypoventilation in individuals with SSS [47,48].

## 4. Discussion

Orofacial manifestations of Down syndrome [49], Marfan syndrome [50], Schwartz–Jampel syndrome [51], Crouzon syndrome [52], Apert syndrome [53], Noonan syndrome [54], and Ellis–Van Creveld syndrome [55] were discussed by many authors. Nevertheless, orofacial manifestations in SSS individuals have not been clearly documented. This review is one of the first attempts to review them comprehensively. The orofacial manifestations and dental anomalies of SSS might occur concomitantly or distinctly along with the common characteristics, and these tend to have consequences. The most frequently reported orofacial manifestations comprise a beaked nose, depressed nasal bridge, enamel hypoplasia, hypodontia, low-set ears, posteriorly rotated ears, deep-set eyes, microcephaly, microdontia, micrognathia, prominent forehead, retrognathia, and thin lips. Accordingly, it is essential that dentists comprehend these characteristics of the clinical appearance of individuals with SSS so as to be dexterous in enabling accurate identification and to develop a treatment plan.

Dental treatment of SSS individuals also poses a challenge to clinicians. Three potential challenges should be assessed and controlled prior to SSS patients under general anesthesia. First, the micrognathic mandible causes difficulty in the airway and also causes difficulty in laryngoscopy and intubation. Therefore, it is prudent to maintain respiration until the airway is safeguarded and bilateral lung ventilation is confirmed [47]. Second, hypocalcemia, hypomagnesemia, hypokalemia, and hyperphosphatemia are common in SSS individuals. This makes it challenging during general anesthesia to maintain electrolytic balance, and this should be suggested as a preoperative correction. The third difficulty encountered, when placing SSS patients under general anesthesia, concerns the chronic use of anticonvulsant drugs in some patients, which alters the metabolism of the anesthetic [44]. In such individuals, dental treatment under general anesthesia is the better option for safe and effective treatment [56]. Postoperative intensive care unit admissions should be considered for individuals with SSS [24,26]. Due to underlying hypocalcemia, SSS patients may also be sensitive to non-depolarizing muscle relaxants. Nonetheless, it is essential to administer a non-depolarizing muscle relaxant, at a low dose, guided by a nerve stimulator [13].

In SSS, the orofacial manifestations are not well documented as typical of the syndrome. Nevertheless, many orofacial abnormalities and dental anomalies also occur in healthy individuals. While this review was established to describe the orofacial and dental manifestations in SSS individuals, they may be merely an association until significant quantities of data are available. However, these manifestations need to be documented and managed, while being aware of the implications caused by the other known features of SSS. This review also establishes the association between dental anomalies (microdontia, hypodontia, high arch palate, and supernumerary teeth) and SSS. Dental treatment including restorations and stainless-steel crowns should be considered for a better prognosis in such individuals [57,58]. It should attempt to enhance the durability of primary teeth by improving dietary habits and mastication, hence promoting the child’s general health and the quality of life in individuals with SSS. Dental practitioners can play a vital role in improving the quality of life for these individuals, through the prevention of dental diseases and the improvement of oral health [59,60]. Pediatric dentists should concentrate on dental health education, improving dietary habits, and early oral evaluation to prevent dental diseases in SSS patients. Furthermore, multidisciplinary management is essential to achieving optimal treatment outcomes [61]. The dynamic role of dental practitioners in increasing the quality of life for these patients through dental disease prevention and oral health improvement in SSS individuals is imperative.

## 5. Conclusions

The most frequently reported orofacial manifestations of SSS comprise beaked nose, depressed nasal bridge, enamel hypoplasia, hypodontia, low-set ears, posteriorly rotated ears, deep-set eyes, microcephaly, microdontia, micrognathia, prominent forehead, retrognathia, and thin lips. This article highlights the orofacial manifestations in individuals with Sanjad–Sakati syndrome. The review paper also establishes the role of general anesthesia in providing dental treatment to SSS individuals. There is a need to establish proper guidelines for the provision of dental treatments in SSS subjects.

## Figures and Tables

**Table 1:** Published case reports on oral facial manifestations in Sanjad–Sakati Syndromic individuals.

Author	Marsden et al.	Al-Ghazali and Dawodu	Al Malik	Platis et al.	Rafique and Al-Yaarubi	Wasersprung et al.	El Batawi	Hafez et al.	Hassona et al.	Ratbi et al.	Prasad et al.
Reference no	17	18	19	20	7	15	13	6	21	10	22
Year	1994	1997	2004	2006	2010	2010	2013	2017	2018	2015	2012
Country	KSA	Oman	KSA	Israel	Oman	Israel	KSA	Egypt	Jordon	Morocco	India
Sex	F	M	F	M	F	1F and 2M	M	3M	F	F	M
Age	5.5Y	9M	4Y	12Y	17Y	11Y, 7Y, 12Y	4Y	9.13Y, 2M, 2Y	15Y	-	6Y
Beaked nose	Y	Y	Y	N	Y	Y	N	Y	Y	Y	Y
Deep set eyes	Y	Y	Y	Y	Y	Y	Y	N	Y	Y	Y
Depressed nasal bridge	Y	Y	Y	Y	Y	Y	Y	N	Y	Y	N
Floppy ear lobes	Y	N	N	Y	Y	Y		Y	Y	Y	N
Frontal bossing	Y	Y	Y	N	N	N	N	Y	Y	N	Y
Hypodontia	N	N	N	N	N	Y	N	N	Y	N	N
Large and low-set years	N	N	Y	Y	Y	N	Y	Y	Y	Y	N
Long philtrum	Y	Y	Y	Y	Y	N	N	Y	N	Y	Y
Micrognathia	Y	Y	Y	Y	N	N	Y	Y	Y	Y	Y
Microcephaly	N	Y	Y	N	Y	Y	N	Y	Y	Y	Y
Microdontia	N	N	N	N	N	Y	Y	N	N	N	N
Supernumerary teeth	N	N	N	N	N	N	Y	N	N	N	N
Thin upper lip	Y	Y	Y	Y	Y	Y	Y	Y	Y	Y	Y

KSA-Kingdom of Saudi Arabia; M-Male; F-Female Y: years, M: Months, D: days; Y-Present; N-Absent or not mentioned.

**Table 2:** Case series studies reported on orofacial manifestations of Sanjad–Sakati Syndrome.

Author	Year	Country	Subjects	Sex	Age	Orofacial Manifestations
Richardson and Kirk [3]	1990	UK	8	Female	0.09Y	Large floppy earlobes, depressed nasal bridge, beaked nose, thin upper lip, long philtrum, and micrognathia
				Female	1.09Y	
				Female	1.2Y	
				Female	1.47Y	
				Male	1.91Y	
				Male	12.8Y	
				Male	3.01Y	
				Male	5.1Y	
Sanjad et al. [1]	1991	Saudi Arabia	12	Male	12D	Prominent forehead, microcephaly, external ear anomalies, beaked nose, depressed nasal bridge, thin lips, deep-set eyes, micrognathia, high arched palate, enamel hypoplasia, and microdontia
				Female	15M	
				Male	1D	
				Female	25D	
				Female	25D	
				Male	2M	
				Male	30D	
				Female	3M	
				Male	4M	
				Female	4M	
				Male	7M	
				Female	9M	
Naguib et al. [23]	2009	Kuwait	21	N	N	posteriorly rotated ears, deep-set eyes; long philtrum; micrognathia; microcephaly;

UK: United Kingdom; Y: years, M: Months, D: days “N“ not mentioned.

## Data Availability

Not applicable.

## References

[1] Sanjad S.A., Sakati N.A., Abu-Osba Y.K., Kaddoura R., Milner R.D. (1991). A new syndrome of congenital hypoparathyroidism, severe growth failure, and dysmorphic features. Arch. Dis. Child..

[2] Touati A., Nouri S., Halleb Y., Kmiha S., Mathlouthi J., Tej A., Mahdhaoui N., Ahmed A.B., Saad A., Bensignor C. (2019). Additional Tunisian patients with Sanjad-Sakati syndrome: A review toward a consensus on diagnostic criteria. Arch. Pediatr..

[3] Richardson R.J., Kirk J.M. (1990). Short stature, mental retardation syndrome, and hypoparathyroidism: A new syndrome. Arch. Dis. Child..

[4] Albaramki J., Akl K., Al Muhtaseb A., Al Shboul M., Mahmoud T., El Khateb M., Hamamy H. (2012). Sanjad Sakati syndrome: A case series from Jordan. East. Mediterr. Health J..

[5] Husain A.Y., Almeshkhas F.M., Hasan Z.A., Hasan M., Isa H.M. (2020). Sanjad Sakati Syndrome. Bahrain Med. Bull..

[6] Hafez M., Anwar G.M., Ibrahim A., Musa N. (2017). Sanjad Sakati syndrome: Case reports from Egypt. Egypt Pediatr. Assoc. Gaz..

[7] Rafique B., Al-Yaarubi S. (2010). Sanjad Sakati syndrome in Omani children. OMJ.

[8] Arabi W.A., Basheer A.A., Abdullah M.A. (2011). Sanjad-Sakati Syndrome in Sudanese children. Sudan J. Paediatr..

[9] Kerkeni E., Sakka R., Sfar S., Bouaziz S., Ghedira N., Ameur K.B., Hmida H.B., Chioukh F.Z., Ghédira E.S., Gribaa M. (2015). Sanjad-Sakati syndrome in a Tunisian child. Arch. Pediatr..

[10] Ratbi I., Lyahyai J., Kabiri M., Banouar M., Zerkaoui M., Barkat A., Sefiani A. (2015). The Bedouin mutation c.155-166del of the TBCE gene in a patient with Sanjad-Sakati syndrome of Moroccan origin. Ann. Saudi Med..

[11] Abuhamda A.F., Elsous A.M. (2020). Sanjad-Sakati syndrome with corneal opacity in a Palestinian neonate: Case report. J. Pediatr. Neonat. Individ. Med..

[12] Sen C., Pal S., Sengupta P., Pal A., Ganguly J., Das C., Basu D. (2016). Sanjad-Sakati syndrome: Beyond the Middle-East. Indian J. Cereb. Palsy.

[13] El Batawi H.Y. (2013). Sanjad-Sakati syndrome Dental Management: A Case Report. Case Rep. Dent..

[14] Padidela R., Kelberman D., Press M., Al-Khawari M., Hindmarsh P.C., Dattani M.T. (2009). Mutation in the TBCE gene is associated with hypoparathyroidism-retardation-dysmorphism syndrome featuring pituitary hormone deficiencies and hypoplasia of the anterior pituitary and the corpus callosum. J. Clin. Endocrinol. Metab..

[15] Bassuni R.I., Kotoury E.L. (2009). Sanjad–Sakati syndrome: A rare autosomal recessive disorder of congenital hypoparathyroidism-microcephaly-mental retardation-seizures-growth retardation. Med. J. Cairo. Univ..

[16] Wasersprung D., Platis C.M., Cohen S., Kaczko L., Zunser I., Peretz B., Katz J. (2010). Case report: Sanjad-Sakati syndrome: Dental findings and treatment. Eur. Arch. Paediatr. Dent..

[17] Marsden D., Nyhan W.L., Sakati N.O. (1994). Syndrome of hypoparathyroidism, growthhormone deficiency, and multiple minor anomalies. Am. J. Med. Genet..

[18] al-Gazali L.I., Dawodu A. (1997). The syndrome of hypoparathyroidism, severe growth failure, developmental delay and distinctive facies. Clin. Dysmorphol..

[19] Al-Malik M.I. (2004). The dentofacial features of Sanjad-Sakati syndrome: A case report. Int. J. Paediatr. Dent..

[20] Platis C.M., Wasersprung D., Kachko L., Tsunzer I., Katz J. (2006). Anesthesia management for the child with Sanjad-Sakati syndrome. Paediatr. Anaesth..

[21] Hassona Y., Rajab L., Taimeh D., Scully C. (2018). Sanjad-Sakati Syndrome: Oral Health Care. Med. Princ. Pract..

[22] Prasad R., Kumari C., Mishra O.P., Singh U.K. (2013). Status epilepticus in a child with Sanjad Sakati syndrome. BMJ Case Rep..

[23] Naguib K.K., Gouda S.A., Elshafey A., Mohammed F., Bastaki L., Azab A.S., Alawadi S.A. (2009). Sanjad-Sakati syndrome/Kenny-Caffey syndrome type 1: A study of 21 cases in Kuwait. East. Mediterr. Health J..

[24] Al Tawil K., Shataiwi A., Mutair A., Eyaid W., Saif S.A. (2005). Hypoparathyroi mism-retardation-dysmorphism (HRD) syndrome in triplets. Am. J. Med. Genet. A.

[25] Kumar K.J., Kumar H.C., Manjunath V.G., Mamatha S. (2013). Hypoparathyroidism-retardation-dysmorphism syndrome. Indian J. Hum. Genet..

[26] Ryabets-Lienhard A., na Ayuthaya A.I., Graham J.M., Pitukcheewanont P. (2018). A Case of Severe TBCE-negative hypoparathyroidism-retardation-dysmorphism syndrome: Case report and literature review. Am. J. Med. Genet..

[27] Aminzadeh M., Galehdari H., Shariati G., Malekpour N., Ghandil P. (2020). Clinical features and tubulin folding cofactor E gene analysis in Iranian patients with Sanjad-Sakati syndrome. J. Pediatr..

[28] Pal K. (2010). Sanjad-Sakati syndrome in a neonate. Indian Pediatr..

[29] Pal K., Moammar H., Mitra D.K. (2010). Visceral myopathy causing chronic intestinal pseudoobstruction and intestinal failure in a child with Sanjad-Sakati syndrome. J. Pediatr. Surg..

[30] Hershkovitz E., Parvari R., Diaz G.A., Gorodischer R. (2004). Hypoparathyroidism-retardation-Dysmorphism (HRD) syndrome—A review. J. Pediatr. Endocrinol. Metab..

[31] Al Dhoyan N., Al Hemidan A.I., Ozand P.T. (2006). Ophthalmic manifestations of Sanjad-Sakati syndrome. Ophthalmic Genet..

[32] Khan A.O., Al-Assiri A., Al-Mesfer S. (2007). Ophthalmic features of hypoparathyroidism-retardation-dysmorphism. J. AAPOS.

[33] Haider A.S., Ganesh A., Al-Kindi A., Al-Hinai A., Al-Kharousi N., Al-Yaroubi S., Al-Zuhaibi S. (2014). New Ocular Associations in Sanjad-Sakati Syndrome: Case report from Oman. Sultan Qaboos Univ. Med. J..

[34] Bouattour N., KamounFeki F. (2020). Neurological Manifestations of Sanjad–Sakati Syndrome: New Three Reported Cases from Tunisia. J. Pediatr. Neurol..

[35] Elhassanien A.F., Alghaiaty H.A. (2013). Neurological manifestations in children with Sanjad-Sakati syndrome. Int. J. Gen. Med..

[36] Alghasab N., Janati A.B., Khan A. (2012). Partial agenesis of corpus callosum in Sanjad-Sakati syndrome (p-ACC). Can. J. Neurol. Sci..

[37] Alomar M.A., Alghafees M.A., Seyam R.M., Aljurayyad A.S., Aldhalaan R.S., Alshuwaier M.K., Alkharashi Y.M., Albassam A.L. (2022). A Staghorn Calcium Phosphate Stone in a Child With Sanjad-Sakati Syndrome: An Iatrogenic Manifestation?. Cureus.

[38] Cader S.H., Shah F.A., Nair S. (2016). Otolaryngologic Manifestations of Sanjad Sakati Syndrome-A Case Report. Otolaryngol. Online J..

[39] Tanna N., Preciado D.A., Biran N. (2009). The otolaryngologic features of Sanjad-Sakati syndrome. Arch. Otolaryngol.-Head Neck Surg..

[40] AlAyed O.A. (2014). Sanjad-Sakati Syndrome and Its Association with Superior Mesenteric Artery Syndrome. Case Rep. Pediatr..

[41] Bashar M., Taimur M., Amreek F., Sayeed K.A., Tahir A. (2020). Endocrinological Manifestations of Sanjad-Sakati Syndrome. Cureus.

[42] Ajarmeh S.A., Al Tamini E.M. (2018). Sanjad-Sakati syndrome with macrocytic anemia and failure to thrive: A case from South Jordan. J. Pediatr. Endocrinol. Metab..

[43] Chinoy A., Skae M., Babiker A., Kendall D., Mughal M.Z., Padidela R. (2017). Impact of intercurrent illness on calcium homeostasis in children with hypoparathyroidism: A case series. Endocr. Connect..

[44] Anteet A.M., Al Issa S.T., Al-Ali A.O., Al-Otaibi H.M., Mohamed S., Babiker A., Al-Jurayyan N.A. (2016). Autoimmune thyroiditis associated with Sanjad-Sakati syndrome: A call for regular thyroid screening. Sudan J. Paediatr..

[45] Hejlesen J., Underbjerg L., Gjørup H., Bloch-Zupan A., Sikjaer T., Rejnmark L., Haubek D. (2018). Dental Findings in Patients with Non-surgical Hypoparathyroidism and Pseudohypoparathyroidism: A Systematic Review. Front. Physiol..

[46] Ahmed M., Sarwani N., Ahmed O. (2019). Sanjad–Sakati syndrome: An anesthetic challenge. J. Bahrain Med. Soc..

[47] Alshoaiby A.N., Rafiq M., Jan R., Shahbaz M., Faqeeh A., Alsohaibani M.A. (2016). Anesthetic management of a case of Sanjad-Sakati syndrome. Saudi J. Anaesth..

[48] Shoback D. (2008). Clinical practice. Hypoparathyroidism. N. Engl. J. Med..

[49] Mubayrik A.B. (2016). The dental needs and treatment of patients with Down syndrome. Dent. Clin. N. Am..

[50] Mallineni S.K., Jayaraman J., Yiu C.K., King N.M. (2012). Concomitant occurrence of hypohyperdontia in a patient with Marfan syndrome: A review of the literature and report of a case. J. Investig. Clin. Dent..

[51] Mallineni S.K., Yiu C.K., King N.M. (2012). Schwartz-Jampel syndrome: A review of the literature and case report. Spec. Care Dent..

[52] Stavropoulos D., Tarnow P., Mohlin B., Kahnberg K.E., Hagberg C. (2012). Comparing patients with Apert and Crouzon syndromes--clinical features and cranio-maxillofacial surgical reconstruction. Swed. Dent. J..

[53] Kreiborg S., Cohen M.M. (1992). The oral manifestations of Apert syndrome. J. Craniofac. Genet. Dev. Biol..

[54] Mallineni S.K., Yung Yiu C.K., King N.M. (2014). Oral manifestations of Noonan syndrome: Review of the literature and a report of four cases. Rom. J. Morphol. Embryol..

[55] Lauritano D., Attuati S., Besana M., Rodilosso G., Quinzi V., Marzo G., Carinci F. (2019). Oral and craniofacial manifestations of Ellis-Van Creveld syndrome: A systematic review. Eur. J. Paediatr. Dent..

[56] Mallineni S.K., Yiu C.K. (2016). Dental treatment under general anesthesia for special-needs patients: Analysis of the literature. J. Investig. Clin. Dent..

[57] Mallineni S.K., Yiu C. (2018). A Retrospective Audit of Dental Treatment Provided to Special Needs Patients under General Anesthesia During a Ten-Year Period. J. Clin. Pediatric Dent..

[58] Tsai C.L., Tsai Y.L., Lin Y.T., Lin Y. (2006). TA retrospective study of dental treatment under general anesthesia of children with or without a chronic illness and/or a disability. Chang. Gung Med. J..

[59] Watt R.G., Williams D.M., Sheiham A. (2014). The role of the dental team in promoting health equity. Br. Dent. J..

[60] Bhatti A., Vinall-Collier K., Duara R., Owen J., Gray-Burrows K.A., Day P.F. (2021). Recommendations for delivering oral health advice: A qualitative supplementary analysis of dental teams, parents’ and children’s experiences. BMC Oral Health.

[61] Taberna M., Gil Moncayo F., Jané-Salas E., Antonio M., Arribas L., Vilajosana E., Peralvez Torres E., Mesía R. (2020). The multidisciplinary team (MDT) approach and quality of care. Front. Oncol..

